# Prognostic Factor Analysis and Nomogram Construction of Primary Retroperitoneal Liposarcoma: A Review of 10 Years of Treatment Experience in a Single Asian Cohort of 211 Cases

**DOI:** 10.3389/fonc.2021.777647

**Published:** 2022-01-12

**Authors:** Aobo Zhuang, Aojia Zhuang, Qian Wu, Weiqi Lu, Hanxing Tong, Yong Zhang

**Affiliations:** ^1^ Department of General Surgery, South Hospital of the Zhongshan Hospital/Shanghai Public Health Clinical Center, Fudan University, Shanghai, China; ^2^ Institute of Biomedical Sciences, Fudan University, Shanghai, China; ^3^ Department of General Surgery, Zhongshan Hospital, Fudan University, Shanghai, China

**Keywords:** Asian, retroperitoneal liposarcoma, primary, prognosis, needle biopsy, progression-free survival (PFS), overall survival (OS), nomogram

## Abstract

**Objective:**

This study intended to retrospectively analyze the data of patients with primary retroperitoneal liposarcoma in a single Asian large-volume sarcoma center and to establish nomograms focused on PRLPS for predicting progression-free survival (PFS) and overall survival (OS).

**Methods:**

A total of 211 patients treated surgically for primary, non-metastatic retroperitoneal liposarcoma during 2009–2021 were identified, and clinicopathologic variables were analyzed. PFS and OS nomograms were built based on variables selected by multivariable analysis. The discriminative and predictive ability of the nomogram was assessed by concordance index and calibration curve.

**Results:**

The median follow-up time was 25 months. A total of 117 (56%) were well-differentiated, 78 (37%) were dedifferentiated, 13 (6%) were myxoid, and 3 (1%) were pleomorphic morphology. Compared to the western population cohort reported by the Memorial Sloan-Kettering Cancer Center, the median age of patients in this cohort was younger (57 vs. 63 years), the tumor burden was lower (20 vs. 26 cm), and the proportion of patients with R0 or R1 resection was higher (97% vs. 81%). The 5-year PFS rate was 49%, and factors independently associated with PFS were symptoms at visit, preoperative needle biopsy, histologic subtypes, and postoperative hospital stay. The 5-year OS rate was 72%. American Society of Anesthesiologists Physical Status and Clavien-Dindo classification were independently associated with OS. The concordance indexes for PFS and OS nomograms were 0.702 and 0.757, respectively. The calibration plots were excellent.

**Conclusions:**

The proposed nomogram provided a favorable reference for the treatment of primary retroperitoneal liposarcoma patients.

## Introduction

Retroperitoneal soft tissue sarcomas (RPS), excluding visceral sarcomas, accounted for 0.15% of all malignancies and approximately 15% of soft tissue sarcomas (1). Surgery is still the cornerstone of all treatments. Complete resection is the only means of radical cure (2). However, the recurrence rate of RPS for 5 years after resection is more than 50% (3, 4). Retroperitoneal liposarcoma is the most common pathological subtype of RPS, accounting for a half of them (5). Previous studies based on large samples of Western populations have shown that histologic subtype and contiguous organ resection are independent risk factors for postoperative recurrence of primary retroperitoneal liposarcoma (6) (PRLPS). The population of Asia exceeds four billion, accounting for more than half of the global population. Previous studies have reported that there may be differences in biological behavior and treatment strategies between eastern and western populations of RLPS (7), but there are very few reports based on Asian populations, and the sample size is small (8, 9).

Several nomograms have been built to optimize the predictive performance of the prognosis of RPS (10–12). However, there is no histological and site-specific nomogram for PRLPS.

Therefore, the purpose of this study is to review a decade-long experience in the treatment of PRLPS in an Asian large-volume sarcoma center, to explore the prognostic factors and to build a prognostic nomogram.

## Methods

### Patients

A 211-patient cohort with curative intent PRLPS between September 2009 and July 2021 at South Hospital of the Zhongshan Hospital/Shanghai Public Health Clinical Center, Fudan University, Shanghai, China, were included. Inclusion and exclusion criteria are as follows: (1) tumor located retroperitoneally, (2) liposarcoma confirmed by pathology, (3) no distant metastasis, (4) no previous surgical resection, and (5) complete follow-up data. This study was approved by the Ethics Committee of South Hospital of Zhongshan Hospital/Shanghai Public Health Clinical Center and carried out in accordance with the Declaration of Helsinki.

### Evaluation of Clinicopathologic Factors

We collected clinicopathological factors, including gender, age at diagnosis, American Society of Anesthesiologists Physical Status (ASA score), symptoms at visit (symptoms), preoperative needle biopsy, tumor location (left or right), tumor burden, histologic subtypes, French Federation of Centers for the Fight against Cancer (FNCLCC) grade, radiation (including preoperative and postoperative), chemotherapy (including preoperative and postoperative), hospital stay, operation (laparoscopic or open), complete resection, surgical procedures, number of combined resections (≤2 or >2), resected organs, operative time, estimated blood loss, packed red blood cell (RBC) transfusion, postoperative intensive care unit (ICU) stay, operative complications [Clavien–Dindo classification (13)], and postoperative hospital stay (POD).

The position of the tumor is determined by the intraoperative assessment of whether the tumor originated on the left or the right. Tumor burden was the sum of the largest diameters of all tumors described in the surgical record. Complete resection is defined as negative margins (R0) or positive micro-margins (R1) but without positive gross margin resection (R2). Abdominal hemi-evisceration is defined as the resection of half-sided abdominal organs. Left hemi-evisceration includes left nephrectomy, left hemicolectomy with/without left adrenal gland resection, pancreatic body tail resection, and spleen resection; right side includes right hemicolectomy, right nephrectomy with/without right adrenal gland resection, cholecystectomy, and partial liver resection.

According to the World Health Organization, liposarcoma is divided into four histologic types: (a) well-differentiated liposarcoma (WDLPS), (b) dedifferentiated liposarcoma (DDLPS), (c) myxoid/round cell liposarcoma (MLPS), and (d) pleomorphic liposarcoma (PLPS) (14). The amplification of oncogenes MDM2 and CDK4 is the standard for the diagnosis of well-differentiated liposarcoma and dedifferentiated liposarcoma; myxoid/round cell liposarcoma is characterized by translocation of FUS and DDIT3 genes, and pleomorphic liposarcoma is diagnosed by the presence of lipoblasts. According to the FNCLCC criteria, LPS was classified into Grade 1, Grade 2, and Grade 3 (14). The role of radiation and chemotherapy in the treatment of PRLPS is still controversial, and only a small number of patients in this study received adjuvant therapy; the status of adjuvant was described only at baseline without further analysis.

### Postoperative Follow-up

Each follow-up requires clinical and imaging examination (CT or MRI form chest to pelvis). Follow-up was required every 3 months for 2 years after operation, then every 6 months, and once a year after 5 years. Disease progression was defined by the radiographic appearance of a new lesion or significant enlargement of the original lesion. Information obtained during follow-up included disease progression and death.

### Statistical Methods

PFS and OS rates were calculated using Kaplan–Meier and compared by log-rank tests. The effect of various clinicopathological factors on OS and PFS was assessed by univariate COX proportional hazard analysis, and variables with p < 0.1 were further included in the multivariate COX model. The independent sample t-test is used to test that the measurement data conform to the normal distribution, and the non-parametric test is used to analyze the non-normal distribution of the measurement data. Statistical data were obtained by χ2 test and Fisher’s exact probability method.

The nomogram was built based on the multivariate Cox model with factors with p < 0.1. Discrimination was assessed using the c-index. For calibration, the patients were divided into 3 subgroups based on the predicted PFS/OS probability, respectively. The mean and 95% confidence interval of each subgroup were calculated and plotted.

All tests were two-tailed, and p < 0.05 was considered statistically significance. All data were analyzed using SPSS 22.0 (SPSS Inc., Chicago, IL, USA) and R 4.0.4 (R Foundation for Statistical Computing, Vienna, Austria; http://www.r-project.org/).

## Results

### Patient and Tumor Characteristics

There were a total of 211 patients who met the enrollment criteria. The median follow-up time was 25 (range, 0.5–140) months. Patient characteristics are listed in **Table 1**. There were 124 (59%) men and 87 (41%) women with a median age of 57 (range, 19–87) years. 68 (32%) patients with preoperative ASA score >2 and 83 (39%) patients had clinical symptoms at the time of consultation. 36 (17%) patients underwent a needle biopsy before surgery (**Table 2**); diagnoses on biopsy and pathology were concordant only in 44% of all cases. The number of tumors located in the left and right sides was basically the same, accounting for 49% and 51%, respectively. The median tumor burden was 20 (range, 2–48) cm. For histologic subtypes, 117 (56%) patients were WDLPS, 78 (37%) were DDLPS, 13 were MLPS (6%), and 3 were for PLPS (1%). There were 75 (36%) cases of FNCLCC grade I, 76 (36%) cases of FNCLCC grade II, and 50 (24%) cases of FNCLCC grade III. 4 (2%) patients received external beam radiation therapy, and 9 (4%) received chemotherapy.

**Table 1:** Patient and tumor characteristics in 211 patients with primary retroperitoneal liposarcoma.

Characteristics	N = 211	% of total
Gender		
Male	124	59
Female	87	41
Age, years median (range)	57	19–87
ASA score		
1–2	143	68
>2	68	32
Symptoms		
Yes	83	39
No	128	61
Needle biopsy		
Yes	36	17
No	175	83
Location		
Left	103	49
Right	108	51
Tumor burden, cm median (range)	20	2–48
Histologic subtypes		
Well-differentiated (WDLPS)	117	56
Dedifferentiated (DDLPS)	78	37
Myxoid/round cell (MLPS)	13	6
Pleomorphic (PLPS)	3	1
FNCLCC		
Grade 1	75	36
Grade 2	76	36
Grade 3	50	24
Unknown	10	4
Radiation		
Yes	4	2
No	207	98
Chemotherapy		
Yes	9	4
No	202	96
Hospital stay, days median (range)	26	5–137

**Table 2:** Comparison of histology between biopsy and pathology.

Histology on pathology	Histology on biopsy					
	WDLPS	DDLPS	MLPS	PLPS	Other	Total
WDLPS	8	1	–	–	2	11
DDLPS	3	5	–	–	13	21
MLPS	–	–	2	–	1	3
PLPS	–	–	–	1	–	1
Total	11	6	2	1	16	36

### Surgical Characteristics

The vast majority of patients underwent open surgery (N = 210), and the tumor was completely removed (97%). For surgical procedures, abdominal hemi-evisceration was performed in 90 (42.7%) patients, only 35 (16.6%) patients had mass resection only, 22 (10.4%) patients had diaphragmatic reconstruction, 14 (6.6) patients had abdominal wall reconstruction, and 13 (6.2%) patients had vascular reconstruction. Of the 90 hemi-evisceration resection patients, 48 (53.3%) were left hemi-evisceration, 17 (35.4%) of which were combined with splenectomy and 12 (25.0%) were combined with pancreatectomy. Major postoperative complications (Clavien–Dindo Classification 3–5) occurred in 10 (20.8%) patients, and the median POD was 17 (range, 6–35) days; 42 (46.7%) were right hemi-evisceration, 3 (7.1%) of which were combined with pancreaticoduodenectomy, major postoperative complications occurred in 5 (11.9%) patients, and the median POD was 20 (range, 5–62) days (**Supplemental Table 1**). 38% of all patients had more than two combined organ resections, and the most common organ resected was colon (57%), followed by kidney (55%) and adrenal gland (20%). The median operative time was 4 (range 1–12) hours. The median estimated blood loss was 500 (range, 20–13,000) ml. Intraoperative packed RBC transfusion was in 66 (31%) patients, with a median transfusion of 4 units. 145 (69%) patients were transferred to the ICU after surgery. Major postoperative complications occurred in 28 (13%) patients, 5 (2.4%) people died within 90 days after surgery, and postoperative mortality at 30, 60, and 90 days was 1.4%, 2.4%, and 2.4%, respectively. The median POD of all patients was 15 (range, 4–109) days (**Table 3**).

**Table 3:** Surgical characteristics in 211 patients with primary retroperitoneal liposarcoma.

Characteristics	N = 211	% of total
Operation		
Laparoscopic surgery	1	0.5
Open surgery	210	99.5
Complete resection		
Yes	205	97
No	6	3
Surgical procedures		
Abdominal hemi-evisceration		
Yes	90	43
No	121	
Mass excision only		
Yes	35	17
No	176	83
Diaphragmatic excision and reconstruction		
Yes	22	10
No	189	90
Abdominal wall excision and reconstruction		
Yes	14	7
No	197	93
Vascular surgery		
Yes	13	6
No	198	94
Gynecologic surgery		
Yes	12	6
No	199	94
Pancreaticoduodenectomy		
Yes	3	1
No	208	99
Number of combined resections		
≤2	130	62
>2	81	38
Resected organs		
Colon	120	57
Kidney	117	55
Adrenal gland	43	20
Spleen	33	16
Pancreas	26	12
Small intestine	26	12
Diaphragm	22	10
Abdominal wall	14	7
Others	44	21
Operative time, hours median (range)	4	1–12
Estimated blood loss, ml median (range)	500	20–13,000
Packed RBC transfusion		
Yes	66	31
No	145	69
Packed RBC transfusion, unit median (range)	4	2–14
ICU stay		
Yes	145	69
No	66	31
ICU stay, days median (range)	4	1–49
Clavien–Dindo classification		
NA	114	54
1–2	69	33
3–5	28	13
Postoperative hospital stay, days median (range)	15	4–109

### Progression-Free Survival Analysis

**Figure 1:**
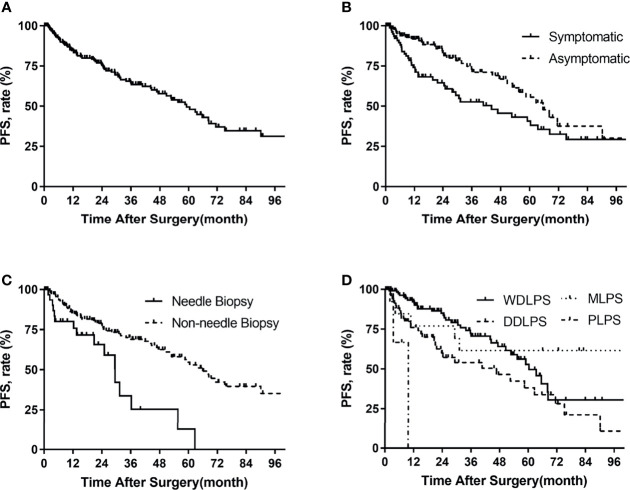
Progression-free survival in patients with primary retroperitoneal liposarcoma by **(A)** all patients, **(B)** symptoms, **(C)** needle biopsy, **(D)** histologic subtypes.

For the 211 patients, 75 (36%) developed progression at the time of last follow-up. The median time to progression was 59 months. The PFS rates were 84% at 1 year, 75% at 2 years, and 49% at 5 years (**Figure 1**). The univariate analysis for prognostic factors of importance to progression is shown in **Table 4**. Compared with asymptomatic patients at the time of consultation, symptomatic patients had a higher risk of disease progression after surgery (p = 0.017) (**Figure 1**). Preoperative needle biopsy was also a risk factor for postoperative disease progression. Specifically, the PFS rates of preoperative biopsy and non-biopsy patients were 84%, 30%, 13% and 85%, 77%, 55% for 1, 3, and 5 years, respectively (**Figure 1**). Histologic subtype was significantly associated with disease progression (p = 0.001). The probability free of progression at 3 years for WDLPS, DDLPS, MLPS, and PLPS was 72%, 54%, 62%, and 0%, respectively (**Figure 1**). FNCLCC grade was also associated with disease progression (p = 0.020). Longer hospital stay, more estimated blood loss, packed RBC transfusion, and longer POD were also related to postoperative disease progression in univariate analysis.

**Table 4:** Univariable and multivariable analyses to determine independent predictors of progression-free survival of primary retroperitoneal liposarcoma.

Variables	Univariate analysis		Multivariate analysis	
	Hazard ratio (95% CI)	p value	Hazard ratio (95% CI)	p value
Gender female vs. male	1.137 (0.715–1.810)	0.587		
Age (continuous)	1.005 (0.985–1.025)	0.634		
ASA score >2 vs. 1–2	1.108 (0.671–1.830)	0.687		
Symptoms yes vs. no	1.752 (1.106–2.776)	0.017	1.866 (1.146–3.041)	0.012
Needle biopsy yes vs. no	2.726 (1.537–4.835)	0.001	2.822 (1.512–5.267)	0.001
Location left vs. right	0.909 (0.576–1.436)	0.683		
Tumor burden (continuous)	1.014 (0.990–1.038)	0.266		
Histologic subtypes		0.001		0.009
DDLPS vs. WDLPS	1.867 (1.152–3.024)		1.559 (0.943–2.577)	
MLPS vs. WDLPS	0.629 (0.242–1.636)		0.491 (0.185–1.303)	
PLPS vs. WDLPS	9.715 (2.228–42.264)		6.281 (1.393–27.760)	
FNCLCC		0.020		
Grade 2 vs. Grade 1	1.588 (0.874–2.884)			
Grade 3 vs. Grade 1	2.544 (1.393–4.646)			
Unknown vs. Grade 1	1.092 (0.321–3.714)			
Complete resection no vs. yes	1.440 (0.804–2.578)	0.220		
Number of combined resections >2 vs. ≤2	1.272 (0.779–2.079)	0.336		
Operative time (continuous)	1.149 (0.969–1.362)	0.110		
Estimated blood loss (continuous)	1.000(1.000–1.000)	0.009	1.000(1.000–1.000)	0.787
Packed RBC transfusion yes vs. no	1.691 (1.058–2.702)	0.028	1.091 (0.586–2.028)	0.784
ICU stay yes vs. no	1.531 (0.915–2.561)	0.105		
Clavien–Dindo classification 3–5 vs. NA/1–2	1.593 (0.722–3.512)	0.245		
Postoperative hospital stay (continuous)	1.031 (1.019–1.04)	<0.001	1.027 (1.014–1.041)	<0.001

Variables with p < 0.1 in the univariate analysis were further included in the multivariate analysis (**Table 4**). In multivariate analysis, symptoms (hazard ratio [HR] 1.866, p = 0.012), needle biopsy (HR 2.822, p = 0.001), histologic subtypes (p = 0.009), and longer POD (HR 1.027, p < 0.001) were independent risk factors for postoperative disease progression.

### Overall Survival Analysis

**Figure 2:**
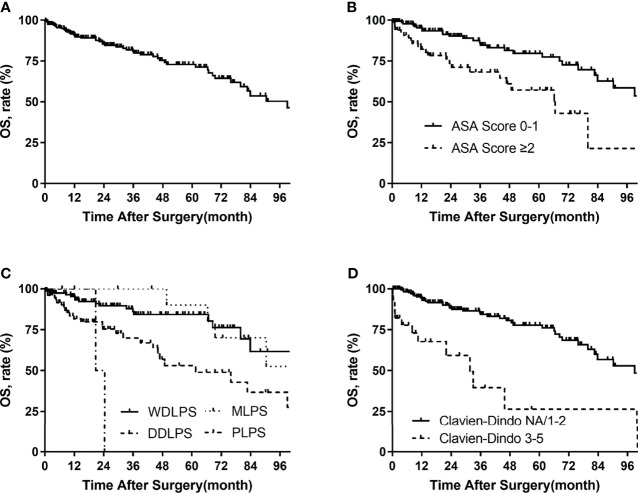
Overall survival in patients with primary retroperitoneal liposarcoma by **(A)** all patients, **(B)** ASA score, **(C)** histologic subtypes, and **(D)** Clavien–Dindo classification.

For the whole group, 49 (23%) patients were dead at the last follow-up. The median overall survival time was 99 months. The overall survival rates were 91% at 1 year, 85% at 2 years, and 72% at 5 years (**Figure 2**). The univariate analysis of risk factors for OS is shown in **Table 5** . The ASA score was prognostic for OS (p = 0.001, **Figure 2**). The 5-year OS of ASA score >2 was 58%, and that of ASA scores 1–2 was 79%. OS for needle biopsy showed a 5-year OS rate of 51% and 76% in biopsies and non-biopsy patients, respectively (**Figure 2** **)**. The probability OS rates at 5 years for well-differentiated, dedifferentiated, myxoid histologic, and pleomorphic subtypes were 85%, 53%, 90%, and 0%, respectively (p = 0.001, **Figure 2**). The correlation between surgical complications and OS is also shown in **Figure 2**, indicating that severe surgical complications (Clavien–Dindo Classification 3–5) affected patients’ OS (p < 0.001). Symptoms (p = 0.039), needle biopsy (0.014), FNCLCC grade (p = 0.007), length of hospital stay (p = 0.015), complete resection (p = 0.038), estimated blood loss (p = 0.011), packed RBC transfusion (p = 0.035), and length of POD (p = 0.009) are also related to OS in univariate analysis.

**Table 5:** Univariable and multivariable analyses to determine independent predictors of overall survival of primary retroperitoneal liposarcoma.

Variables	Univariate analysis		Multivariate analysis	
	Hazard ratio (95% CI)	p value	Hazard ratio (95% CI)	p value
Gender female vs. male	1.148 (0.650–2.027)	0.635		
Age (continuous)	1.019 (0.995–1.045)	0.123		
ASA score >2 vs. 1–2	2.633 (1.466–4.728)	0.001	2.293 (1.221–4.308)	0.010
Symptoms yes vs. no	1.872 (1.032–3.235)	0.039	1.417 (0.751–2.676)	0.282
Needle biopsy yes vs. no	2.367 (1.195–4.689)	0.014	1.946 (0.861–4.399)	0.110
Location left vs. right	1.429 (0.805–2.539)	0.223		
Tumor burden (continuous)	1.011 (0.982–1.041)	0.458		
Histologic subtypes		0.001		0.056
DDLPS vs. WDLPS	2.953 (1.583–5.511)		1.893 (0.672–5.337)	
MLPS vs. WDLPS	1.075 (0.356–3.250)		1.125 (0.309–4.095)	
PLPS vs. WDLPS	10.402 (2.328–46.486)		9.815 (1.792–53.750)	
FNCLCC		0.007		0.781
Grade 2 vs. Grade 1	1.690 (0.766–3.729)		0.942 (0.116–7.623)	
Grade 3 vs. Grade 1	3.426 (1.606–7.308)		1.510 (0.157–14.544)	
Unknown vs. Grade 1	0.830 (0.105–6.539)		0.997 (0.113–8.809)	
Complete resection no vs. yes	1.876 (1.035–3.401)		1.176 (0.557–2.486)	0.671
Number of combined resections >2 vs. ≤2	1.109 (0.899–1.368)	0.333		
Operative time (continuous)	1.020 (0.793–1.311)	0.880		
Estimated blood loss (continuous)	1.000 (1.000–1.000)	0.011	1.000 (1.000–1.000)	0.905
Packed RBC transfusion yes vs. no	1.860 (1.044–3.313)	0.035	1.022 (0.461–2.263)	0.958
ICU stay yes vs. no	1.699 (0.883–3.271)	0.113		
Clavien–Dindo classification 3–5 vs. NA/1–2	4.663 (2.391–9.094)	<0.001	3.648 (1.521–8.275)	0.003
Postoperative hospital stay (continuous)	1.019 (1.005–1.033)	0.009	1.001 (0.985–1.107)	0.943

Multivariate Cox analysis showed that the following factors were significantly associated with OS: ASA score >2 vs. 1–2 (HR 2.293, p = 0.010) and Clavien–Dindo classification 3–5 vs. NA/1–2 (HR 3.648, p = 0.003).

### Development and Validation of the Nomogram Prediction Model

**Figure 3:**
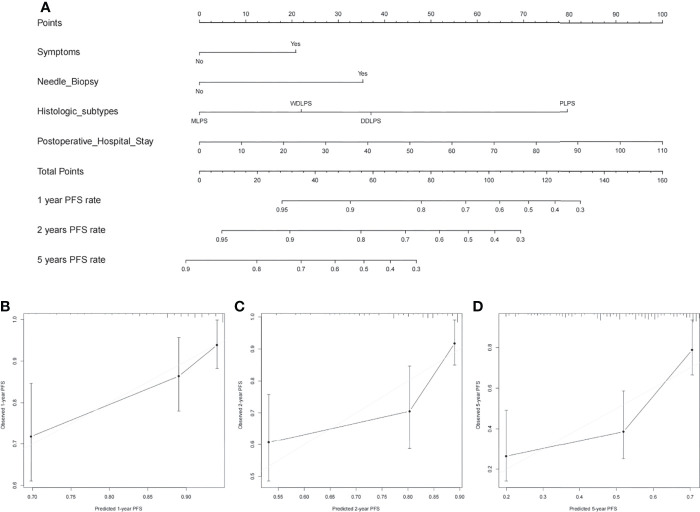
**(A)** Nomogram for 1-, 2-, and 5-year progression-free survival in patients with primary retroperitoneal liposarcoma and calibration plots for internal validation of **(B)** 1-, **(C)** 2-, and **(D)** 5-year progression-free survival nomogram.

**Figure 4:**
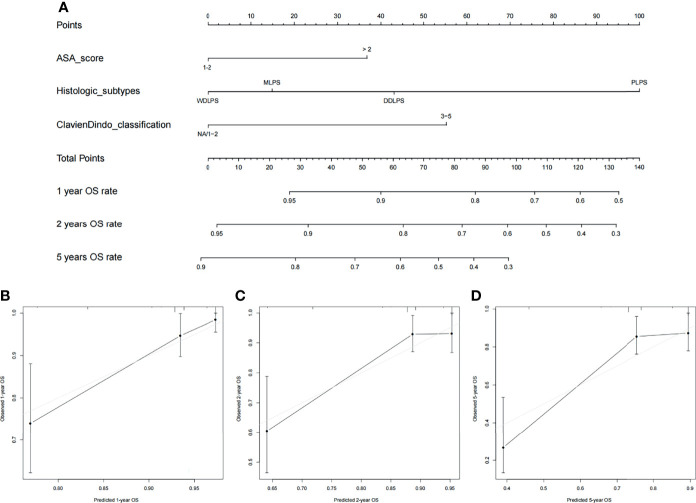
**(A)** Nomogram for 1-, 2-, and 5-year overall survival in patients with primary retroperitoneal liposarcoma and calibration plots for internal validation of **(B)** 1-, **(C)** 2-, and **(D)** 5-year overall survival nomogram.

Nomograms were built to predict PFS and OS at 1, 2, and 5 years (**Figures 3**, **4**). The calibration plots (**Figures 3**, **4**) demonstrate good agreement between the nomogram predictions and the actual outcomes. The concordance indices and bootstrapped 95% confidence intervals were 0.702 (0.687–0.850) for the PFS nomogram and 0.757 (0.631–0.883) for the OS nomogram.

## Discussion

This study was based on a single-center cohort of 211 cases of PRLPS in an Asian center. The preoperative characteristics, surgical status, and short-term prognosis were comprehensively analyzed, as well as the risk factors of PFS and OS in patients with PRLPS. Moreover, two histology-specific and site-specific nomograms were established. Compared with the western population cohort reported by the Memorial Sloan-Kettering Cancer Center (6), the median age of patients in this cohort was younger (57 vs. 63 years), the tumor burden was lower (20 vs. 26 cm), and the proportion of patients with R0 or R1 resection was higher (97% vs. 81%). As for the sex ratio, the proportion of adjuvant therapy and the OS rate for 5 years was roughly the same. Compared with a series of over 1,000 patients with primary RPS, treated at 8 European/North American sarcoma reference centers reported by the Trans-Atlantic RPS Working Group (3), there were no significant differences in gender, age, tumor burden, and FNCLCC grade. In terms of completeness of surgical resection ratio and surgical complications ratio, the ratio of patients receiving radiotherapy and chemotherapy in this cohort was significantly lower. However, there was no significant difference in the proportion of recurrence and overall survival at 5 years after surgery.

Since the retroperitoneal space is asymptomatic, the RLPS may already be large at the time of diagnosis and is usually detected during an accidental or scheduled physical examination. The reported clinical symptoms are principally abdominal pain and distension (8). In our study, 39% of patients with PRLPS were symptomatic. Compared with asymptomatic patients, patients with symptoms had a nearly two-fold increased risk of disease progression after surgery. This may be related to a higher growth rate, which in turn may be related to biological aggressiveness. As far as we know, we report for the first time that the presence or absence of symptoms is an independent risk factor for postoperative PRLPS progression.

In the analysis of prognostic factors, we also found that the pathological type is an independent prognostic factor for PFS (p = 0.009) and is also closely related to OS (p = 0.056). Patients with DDLPS have a clearly worse outcome than those with an entire WDLP (15, 16). Specifically, patients with DDLPS experience locoregional recurrence sooner and more frequently compared with those with only WD. Importantly, DD patients (but not WD patients) have a moderate likelihood of distant metastasis (up to 30%–40%), usually to the lungs (3). Therefore, disease biology is still the “King” in PRLPS.

Multivariate analysis suggested that the risk of disease progression after preoperative puncture patients increased twice (p =0.001). However, previous studies put forward the view that preoperative core needle biopsy for RPS is safe and does not affect oncological outcome (17, 18). In an in-depth review of our data again, we found that there are two reasons for the above deviations: First, in this study, the overall diagnostic accuracy of 36 biopsy cases was only 44% (16/36). A wrong preoperative diagnosis may lead to wrong treatment results and worse prognosis. In needle biopsy, non-diagnostic samples can arise due to technical errors and the histological heterogeneity of tumors (19). In this cohort, the diagnosis of puncture pathology in most patients with DDLPS was incorrect or inadequate because of their heterogeneity; that is, the degree of differentiation of different tumor parts was different. Therefore, areas that appear to be of a higher tumor grade on cross-sectional imaging should be biopsied, and multiple cores using ≥14-gauge needles from different tumor locations and depths in correlation with radiological imaging should be performed according to the current guidelines (20). Another possible reason for the poor prognosis was the choice of biopsy patients. It is shown in **Supplementary Table 1** that compared with non-biopsy patients, biopsy patients had a higher tumor grade, more combined organ resection, longer operation time, intraoperative bleeding, and postoperative ICU stay (**Supplemental Table 2**), indicating that the attending physician performed a preoperative biopsy on tumor patients with more aggressive clinical behaviors. Therefore, there may be a selection bias.

In multivariate analysis, we found for the first time that POD was associated with a higher recurrence of PRLPS (<0.001). We then divided POD into the long-POD group (POD >15 days) and short-POD group (POD ≤15 days) by the median of 15 days. We found that the tumor burden in the long hospital stay group was significantly higher (p = 0.015), with more organ resections (0.016), the operation time was longer (<0.001), and the proportion of packed RBC transfusion was also higher (p = 0.007) (**Supplemental Table 3**). Based on the above differences, we speculated that the reasons why prolonged POD related to the recurrence of the disease are as follows. First, as Lahat et al. hypothesized, aggressive tumors may require more aggressive resection (such as DDLPS), while for relatively indolent tumors such as WDLPS, a satisfactory treatment effect can be achieved by ensuring that the margin is negative (16). Therefore, it can be speculated that the longer POD reflects more complicated surgery, which in turn may reflect more advanced and/or aggressive tumors with poor prognosis. Secondly, blood transfusion may increase the risk of recurrence through transfusion-related immunosuppression (21, 22). Transfusions increase suppressor T cell activity and inhibit natural killer cell activity, and the mitogenic activity of platelet-derived growth factors increases during storage of blood and may stimulate tumor growth following transfusion (23). Therefore, perioperative transfusion may stimulate tumor growth directly or by an immunosuppressive effect, thereby having an adverse effect on patient prognosis.

Several nomograms have been built to optimize the predictive performance of the PFS and OS of retroperitoneal sarcoma (4, 10, 24–26). Gronchi and colleagues presented an externally validated nomogram to predict the likelihood of RPS for 7-year OS and DFS in 2013 and was specifically recommended by the AJCC (12). Marcus et al. also established nomograms to predict disease-specific death, local recurrence, and distant recurrence at 3, 5, and 15 years in 2016 (4). However, there is no histology-specific and site-specific nomogram focused on PRLPS only. In this study, we observed that for patients with PRLPS who underwent surgical resection, the crucial prognostic factors for PFS were the symptoms, needle biopsy, histologic subtypes, and postoperative length of hospital stay. ASA score and Clavien–Dindo classification were the factors that affected OS. Based on the factors mentioned above, we developed two nomograms to predict the PFS and OS of patients with PRLPS after surgery. The AUC of the two nomograms both exceeds 0.7, and the calibration plots demonstrate good agreement between the predictions made by the nomograms and the actual outcomes. PRLPS is a relatively rare disease, and a large number of patients have undergone primary resection in local hospitals. This study can provide a reference for postoperative consultations for those centers with relatively inexperienced diagnosis and treatment. Secondly, this study predicts the PFS and OS of patients at 1, 2, and 5 years after surgery, which provides a useful reference for the formulation of postoperative follow-up plans. Furthermore, this study is the first PRLPS cohort study based on an Asian population, which provides reference for the treatment of such rare disease in the Asian population. Therefore, we believe that these two nomogram prediction models for PRLPS can help doctors in clinical decision-making.

This work also had several limitations. Firstly, as a retrospective study, there may be missing data, recall bias, and errors in the initial medical records. Secondly, the study was a review of nearly 10 years of treatment experience, with a median follow-up of only 25 months, which needs to be extended further to provide more reliable data. Third, while the performance of this nomogram was verifiably useful in our cohort, external validation with PRLPS patients from other institutions is still needed.

In summary, this study reviewed the treatment experience of 211 patients with primary retroperitoneal liposarcoma in an Asian large-volume sarcoma center for 10 years. The preoperative data, surgical data, and postoperative prognostic factors of patients were analyzed, the risk factors of postoperative PFS and OS were explored, and nomogram prediction models were established, so as to provide evidence for the diagnosis and treatment of primary retroperitoneal liposarcoma in the Asian population.

## Data Availability Statement

The raw data supporting the conclusions of this article will be made available by the authors, without undue reservation.

## Ethics Statement

The studies involving human participants were reviewed and approved by the Ethics Committee of South Hospital of Zhongshan Hospital/Shanghai Public Health Clinical Center. The patients/participants provided their written informed consent to participate in this study.

## Author Contributions

ABZ, AJZ, and QW collected, analyzed, and interpreted the patient data. ABZ was a major contributor in writing the manuscript. WL provided writing ideas and helped in data analysis and article proofreading. HT and YZ provided the research ideas and guidance. All authors contributed to the article and approved the submitted version.

## Conflict of Interest

The authors declare that the research was conducted in the absence of any commercial or financial relationships that could be construed as a potential conflict of interest.

## Publisher’s Note

All claims expressed in this article are solely those of the authors and do not necessarily represent those of their affiliated organizations, or those of the publisher, the editors and the reviewers. Any product that may be evaluated in this article, or claim that may be made by its manufacturer, is not guaranteed or endorsed by the publisher.
