# Supplementary material for: Prognostic Factor Analysis and Nomogram Construction of Primary Retroperitoneal Liposarcoma: A Review of 10 Years of Treatment Experience in a Single Asian Cohort of 211 Cases
Source: Front Oncol. 2022 Jan 12;11:777647. doi: 10.3389/fonc.2021.777647 (PMC8790090; doi:10.3389/fonc.2021.777647)
Supplement: Supplementary file 1 [file Table_1.docx]

Supplemental Table 1 Sugical characteristics in 90 patients with abdominal hemi-evisceration.

| Characteristics | left hemi-evisceration (N=48) | right hemi-evisceration (N=42) |
| --- | --- | --- |
| Operation |  |  |
| Laparoscopic Surgery | 0 | 0 |
| Open Surgery | 48 | 42 |
| Complete Resection |  |  |
| Yes | 48 | 42 |
| No | 0 | 0 |
| Surgical Procedures |  |  |
| Diaphragmatic Reconstruction | 4 | 8 |
| Abdominal Wall Reconstruction | 1 | 4 |
| Vascular Surgery | 2 | 4 |
| Gynecologic Surgery | 3 | 2 |
| Pancreaticoduodenectomy | 0 | 3 |
| Number Of Combined Resections |  |  |
| ≤2 | 12 | 18 |
| >2 | 36 | 24 |
| Resected Organs |  |  |
| Colon | 48 | 42 |
| Kidney | 48 | 42 |
| Adrenal gland | 19 | 17 |
| Spleen | 17 | 0 |
| Pancreas | 12 | 3 |
| Small Intestine | 3 | 4 |
| Diaphragm | 4 | 8 |
| Abdominal Wall | 1 | 4 |
| Operative time, hours median (range) | 4 (2.5-8.5) | 4 (2-8) |
| Estimated blood loss, ml median (range) | 500 (50-6000) | 550 (100-2500) |
| Packed RBC transfusion |  |  |
| Yes | 19 | 14 |
| No | 29 | 28 |
| Packed RBC transfusion, unit median (range) | 3 (2-6) | 3 (2-6) |
| ICU Stay |  |  |
| Yes | 36 | 33 |
| No | 12 | 9 |
| ICU Stay, days median (range) | 4 (1-35) | 5 (1-45) |
| Clavien–Dindo classification |  |  |
| NA | 24 | 20 |
| 1-2 | 14 | 17 |
| 3-5 | 10 | 5 |
| Postoperative Hospital Stay, days median (range) | 17 (6-35) | 20 (5-62) |

Supplemental Table 2 characteristics for needle biopsy and non-needle biopsy patients.

| Characteristics | Needle Biopsy (N=36) | Non-needle Biopsy (N=175) | p |
| --- | --- | --- | --- |
| Gender |  |  | 0.754 |
| Male | 22 | 102 |  |
| Female | 14 | 73 |  |
| Age, years median (range) | 58 (33-77) | 56 (19-87) | 0.976 |
| ASA score |  |  | 0.814 |
| 1-2 | 25 | 118 |  |
| >2 | 11 | 57 |  |
| Symptoms |  |  | 0.664 |
| Yes | 13 | 70 |  |
| No | 23 | 105 |  |
| Location |  |  | 0.601 |
| Left | 19 | 84 |  |
| Right | 17 | 91 |  |
| Tumor burden, cm median (range) | 20 (2-48) | 20 (4-45) | 0.761 |
| Histologic subtypes |  |  | 0.011 |
| Well-diferentiated (WDLPS) | 11 | 106 |  |
| Dediferentiated (DDLPS) | 21 | 57 |  |
| Myxoid/Round cell (MLPS) | 3 | 10 |  |
| Pleomorphic (PLPS) | 1 | 2 |  |
| FNCLCC |  |  | 0.036 |
| Grade 1 | 8 | 67 |  |
| Grade 2 | 14 | 62 |  |
| Grade 3 | 14 | 36 |  |
| Unknown | 0 | 10 |  |
| Radiation |  |  | 0.136 |
| Yes | 2 | 2 |  |
| No | 34 | 173 |  |
| Chemotherapy |  |  | <0.001 |
| Yse | 7 | 2 |  |
| No | 29 | 173 |  |
| Hospital Stay, days median (range) | 30 (5-81) | 24 (7-137) | 0.285 |
| Operation |  |  | 1.000 |
| Laparoscopic Surgery | 0 | 1 |  |
| Open Surgery | 36 | 174 |  |
| Complete Resection |  |  | 0.104 |
| Yes | 33 | 172 |  |
| No | 3 | `3 |  |
| Surgical Procedures |  |  |  |
| Half-sided Abdominal Evisceration | 15 | 75 | 0.895 |
| Mass Excision Only | 3 | 33 | 0.217 |
| Diaphragmatic Reconstruction | 3 | 19 | 0.879 |
| Abdominal Wall Reconstruction | 3 | 11 | 0.653 |
| Vascular Surgery | 6 | 7 | 0.002 |
| Gynecologic Surgery | 3 | 9 | 0.721 |
| Pancreaticoduodenectomy | 0 | 3 | 0.985 |
| Number Of Combined Resections |  |  | 0.116 |
| ≤2 | 18 | 112 |  |
| >2 | 18 | 63 |  |
| Resected Organs |  |  |  |
| Colon | 26 | 94 | 0.041 |
| Kidney | 18 | 99 | 0.470 |
| Adrenal gland | 6 | 37 | 0.544 |
| Spleen | 10 | 23 | 0.028 |
| Pancreas | 8 | 18 | 0.047 |
| Small Intestine | 9 | 17 | 0.011 |
| Diaphragm | 3 | 19 | 0.879 |
| Abdominal Wall | 3 | 11 | 0.935 |
| Operative time, hours median (range) | 4.5 (1-12.5) | 4 (1.5-8.5) | 0.009 |
| Estimated blood loss, ml median (range) | 700 (50-13000) | 400 (20-6000) | <0.001 |
| Packed RBC transfusion |  |  | <0.001 |
| Yes | 45 | 21 |  |
| No | 130 | 15 |  |
| Packed RBC transfusion, unit median (range)* | 4 (2-14) | 4 (1-8) | 0.140 |
| ICU Stay |  |  | 0.009 |
| Yes | 114 | 32 |  |
| No | 61 | 4 |  |
| ICU Stay, days median (range)* | 5 (2-49) | 4 (1-45) | 0.005 |
| Clavien–Dindo classification |  |  | 0.231 |
| NA/1-2 | 154 | 29 |  |
| 3-5 | 21 | 7 |  |
| Postoperative Hospital Stay, days median (range) | 20 (4-58) | 15 (5-109) | 0.285 |

Supplemental Table 3 characteristics for long-postoperative hospital stay group and short-postoperative hospital stay group patients.

| Characteristics | long-postoperative hospital stady (N=99) | short-postoperative hospital stay (N=112) | p |
| --- | --- | --- | --- |
| Gender |  |  | 0.610 |
| Male | 60 | 64 |  |
| Female | 39 | 48 |  |
| Age, years median (range) | 58 (31-85) | 56 (19-87) | 0.326 |
| ASA score |  |  | 0.798 |
| 1-2 | 68 | 75 |  |
| >2 | 31 | 37 |  |
| Symptoms |  |  | 0.252 |
| Yes | 43 | 40 |  |
| No | 56 | 72 |  |
| Location |  |  | 0.311 |
| Left | 52 | 51 |  |
| Right | 47 | 61 |  |
| Tumor burden, cm median (range) | 22 (2-42) | 20 (4-48) | 0.015 |
| Histologic subtypes |  |  | 0.292 |
| Well-diferentiated (WDLPS) | 55 | 62 |  |
| Dediferentiated (DDLPS) | 39 | 39 |  |
| Myxoid/Round cell (MLPS) | 3 | 10 |  |
| Pleomorphic (PLPS) | 2 | 1 |  |
| FNCLCC |  |  | 0.687 |
| Grade 1 | 33 | 42 |  |
| Grade 2 | 35 | 41 |  |
| Grade 3 | 27 | 23 |  |
| Unknown | 2 | 5 |  |
| Radiation |  |  | 0.343 |
| Yes | 3 | 1 |  |
| No | 96 | 111 |  |
| Chemotherapy |  |  | 0.737 |
| Yse | 5 | 4 |  |
| No | 94 | 108 |  |
| Operation |  |  | 1.000 |
| Laparoscopic Surgery | 0 | 1 |  |
| Open Surgery | 99 | 110 |  |
| Complete Resection |  |  | 0.423 |
| Yes | 95 | 110 |  |
| No | 4 | 2 |  |
| Surgical Procedures |  |  |  |
| Half-sided Abdominal Evisceration | 50 | 40 | 0.030 |
| Mass Excision Only | 9 | 26 | 0.009 |
| Diaphragmatic Reconstruction | 13 | 9 | 0.227 |
| Abdominal Wall Reconstruction | 10 | 4 | 0.057 |
| Vascular Surgery | 10 | 3 | 0.041 |
| Gynecologic Surgery | 8 | 4 | 0.234 |
| Pancreaticoduodenectomy |  |  |  |
| Number Of Combined Resections |  |  | 0.016 |
| ≤2 | 47 | 34 |  |
| >2 | 52 | 78 |  |
| Resected Organs |  |  |  |
| Colon | 70 | 50 | <0.001 |
| Kidney | 60 | 57 | 0.157 |
| Adrenal gland | 22 | 21 | 0.778 |
| Spleen | 24 | 9 | 0.001 |
| Pancreas | 19 | 7 | 0.004 |
| Small Intestine | 11 | 15 | 0.615 |
| Diaphragm | 13 | 9 | 0.227 |
| Abdominal Wall | 10 | 4 | 0.057 |
| Operative time, hours median (range) | 4 (1-12) | 3.6 (1.5-7.3) | <0.001 |
| Estimated blood loss, ml median (range) |  |  | 0.006 |
| Packed RBC transfusion |  |  | 0.007 |
| Yes | 40 | 26 |  |
| No | 59 | 86 |  |
| Packed RBC transfusion, unit median (range) | 500 (4-13000) | 300 (20-6000) | 0.062 |
| ICU Stay |  |  | <0.001 |
| Yes | 81 | 65 |  |
| No | 18 | 47 |  |
| Clavien–Dindo classification |  |  | 0.001 |
| NA/1-2 | 78 | 105 |  |
| 3-5 | 21 | 7 |  |
